# Characterization of the anti-AChE potential and alkaloids in Rhizoma Coptidis from different *Coptis* species combined with spectrum-effect relationship and molecular docking

**DOI:** 10.3389/fpls.2022.1020309

**Published:** 2022-10-31

**Authors:** Luming Qi, Furong Zhong, Nannan Liu, Jie Wang, Kaidi Nie, Youli Tan, Yuntong Ma, Lina Xia

**Affiliations:** ^1^ School of Health Preservation and Rehabilitation, Chengdu University of Traditional Chinese Medicine, Chengdu, China; ^2^ State Administration of Traditional Chinese Medicine Key Laboratory of Traditional Chinese Medicine Regimen and Health, Chengdu University of Traditional Chinese Medicine, Chengdu, China; ^3^ School of Pharmacy, Chengdu University of Traditional Chinese Medicine, Chengdu, China; ^4^ Department of Pharmacy, Affiliated Sport Hospital of CDSU, Chengdu Sport University, Chengdu, China

**Keywords:** Rhizoma Coptidis, *Coptis* species, alkaloids, AChE inhibition, Spectrum-effect relationship, Molecular docking

## Abstract

*Coptis* species are the main source of Rhizoma Coptidis (RC) drugs, which have always been used to treat Alzheimer’s disease in the clinical experience of ancient China. However, many species of this genus have been largely underutilized until now. With this fact, this research has been designed to investigate for the first time the anti-acetylcholinesterase (AChE) property of different extracts for RC drugs from four *Coptis* species (*C. chinensis*, *C. deltoidea*, *C. teeta* and *C. omeiensis*) and to quantify the main alkaloids. Petroleum ether, ethyl acetate and n-butanol fractions of RC drugs were sequentially collected using an accelerated solvent extraction technique. Spectrum-effect relationship and molecular docking were applied to analyse the relationships between alkaloids and AChE inhibitory activity. The N-butanol extract was proven to be the main active fraction, and *C. teeta* may be the best source of RC drugs for Alzheimer’s disease treatment, with significantly lower IC 20, IC 50 and IC 80 values for AChE inhibition. The UPLC/QqQ-MS quantitative analysis showed that the accumulations of 10 alkaloids in RC drugs from different sources greatly varied. Three data processing methods (Random forest, Boruta and Pearson correlation) comprehensively analysed the spectrum-effect relationship and revealed that columbamine, berberine and palmatine were the most important AChE inhibitors that could be used as quality markers to select RC drugs for Alzheimer’s disease treatment. In addition, the dominant compounds were successfully docked against AChE to verify the binding affinity and interactions with the active site. The present study can contribute to the reasonable development and utilization of RC drugs from different sources, especially to provide certain evidence for their application in the treatment of Alzheimer’s disease.

## Introduction

Alzheimer’s disease is the most common age-related disease with chronic memory and cognitive decline as the main manifestations, followed by psychiatric symptoms, behavioural disorders and impairment of activities in daily lives (Ju and Tam, 2022). With the increasing ageing of the world, the incidence rate of this disease continues to increase, which has attracted extensive attention from scientific researchers. Acetylcholinesterase (AChE) inhibitors, including tacrine hydrochloride, huperzine A, and donepezil, are currently used drugs to relieve the symptoms of this disease (Taqui et al., 2022). Although these drugs can delay disease progression, most of them are accompanied by certain side effects (Refaay et al., 2022) Therefore, the screening of AChE inhibitors from natural products has become a research hotspot to better treat Alzheimer’s disease (Lin et al., 2020b; Ahmed et al., 2021).

The root and rhizome material of the *Coptis* species Rhizoma Coptidis (RC) is a top-grade drug that has been documented to have an excellent effect on improving memory and treating brain diseases in ancient clinical experience in China. Modern pharmacological studies have proven that this drug has a positive therapeutic potential on Alzheimer’s disease, and several alkaloids have been identified as the main AChE inhibitors (Zhao et al., 2016; Cao et al., 2018; Lin et al., 2020a). However, these studies mainly focused on the extract of RCdrugs from *C. chinensis* Franch, and other species of the *Coptis* genus have never been investigated regarding their anti-AChE properties. In particular, some studies have demonstrated that RC drugs separated from different *Coptis* plants always have different therapeutic effects. For example, Feng et al. (Feng et al., 2011) compared the antibacterial effect of different RC drugs and demonstrated that drugs from *C. chinensis* had the highest inhibitory effect against *Staphylococcus aureus*. Our research team has proven that RC drugs from *C. deltoidea* have a better hypoglycaemic effect than those from the other three *Copti*s species (Chen et al., 2021). In summary, the anti-AChE potential of RC drugs from different *Coptis* species has never been investigated, and the relationships between this property and alkaloids are unclear.

Because natural medicinal materials are subject to a series of metabolic processes that produce multipolar chemical profiles, an efficient extraction method is a key step for their chemical and pharmacological analysis (Vezzulli et al., 2022). Accelerated solvent extraction techniques with the advantages of easy operation, high speed and efficiency are more effective for pre-treating these complex biological systems. Based on an efficient preparation procedure, the activity assay and chemical analysis can be more accurately achieved. In addition, spectrum-effect relationship analysis and molecular docking are popular methods to analyse the association between chemical compounds and the biological activity of natural products (Shi et al., 2016; da Silva Barbosa et al., 2020; Chang et al., 2021; Lu et al., 2022; Amir Rawa et al., 2022). Spectrum-effect relationship analysis refers to constructing the relationship of the chemical profiles with specific biological activity and this association can be used to reflect the internal quality of natural drugs (Zhang et al., 2018). Molecular docking is a commonly used technique based on structural molecular biology and computer-assisted drug design that can effectively predict the binding mechanism of a ligand with a protein based on the three-dimensional structure (Mtemeli et al., 2022). These techniques have been proven effective for the in-depth analysis of the association mechanism between biological activity and chemical compositions regarding natural drugs.

Thus, the present study was designed to investigate the anti-AChE potential of n-butanol, ethyl acetate and petroleum ether extracts of RC drugs from four *Coptis* species (*C. chinensis*, *C. deltoidea*, *C. omeiensis* and xtitC. teeta) based on an *in vitro* activity assay and to quantify the main alkaloids in the active fraction using ultra-performance liquid chromatography with triple quadrupole mass spectrometry (UPLC/QqQ-MS). The accelerated solvent extraction technique was used to obtain different extraction fractions. Furthermore, spectrum-effect relationships and molecular docking techniques were jointly applied in an auxiliary manner to analyse the internal association between alkaloid profiles and anti-AChE activity. We hope that our conclusion can provide an effective foundation for the rational application of RC drugs from different *Coptis* species, especially for the treatment of Alzheimer’s disease.

## Materials and methods

### RC drugs materials

Four *Coptis* species *C. chinensis*, *C. deltoidea*, *C. omeiensis* and *C. teeta* were collected in their harvest time (n=6). The sampling site of first three plants is Heishan Village, Hongya County, Sichuan Province of China (Longitude: 103.1611, Latitude: 29.5097), and the sampling site of *C. teeta* is Pihe Township, Fugong County, Yunnan Province of China (Longitude: 98.9236, Latitude: 26.5375). All materials were collected from 5-year-old plants which were identified by Pro. Ma of Chengdu University of Traditional Chinese Medicine.

**Figure 1:**
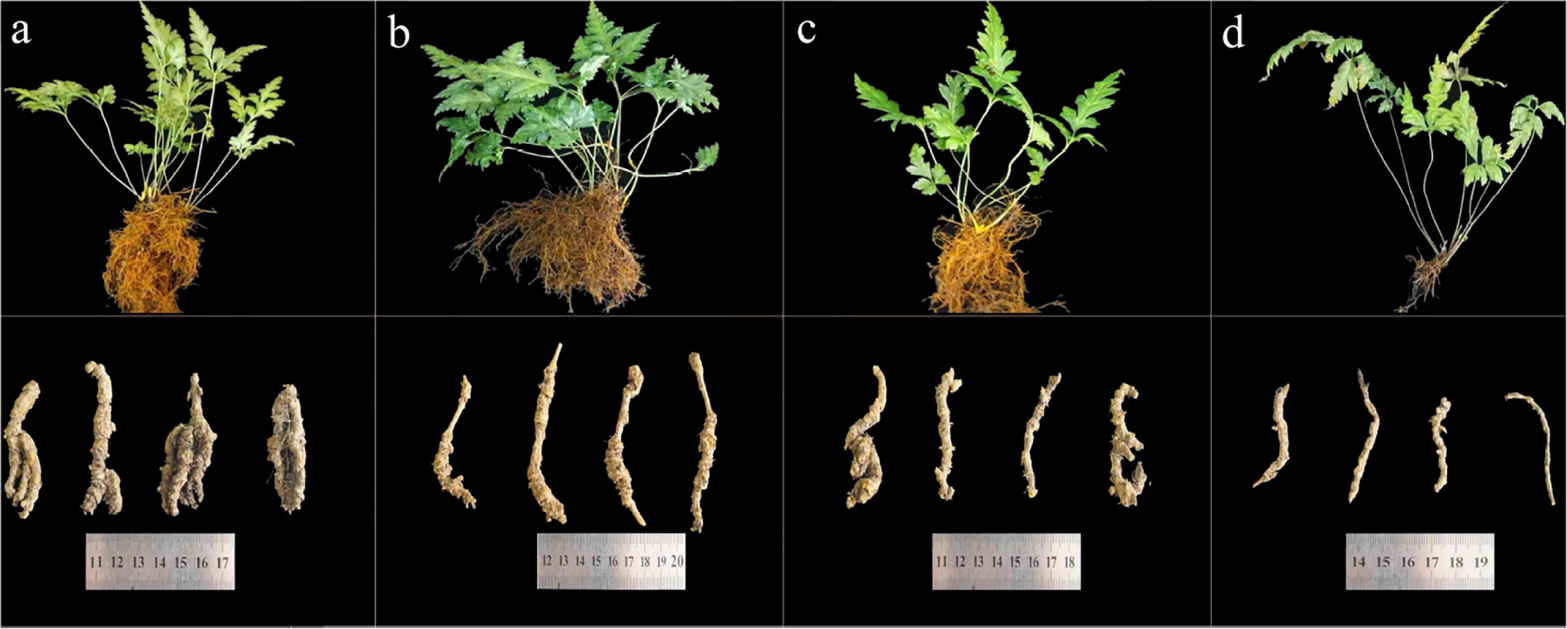
The images of RC materials. **(A)** *C. chinensis* species and its root and rhizome; **(B)** *C. deltoidea* species and its root and rhizome; **(C)** *C. omeiensis* species and its root and rhizome; **(D)** *C. teeta* species and its root and rhizome.).

Roots and rhizomes of these plants were firstly separated and washed by deionized water. Then, these materials were dried in an oven at a 60°C condition and smashed using a pulverizer. The obtained powder were filtered through a 100-mesh sieve. Finally, these RC drugs were labelled and stored in a cool condition for the subsequent analysis. The images of these materials are presented in **Figure 1**.

### Reagents

Chemical standards of magnoflorine, groenlandicine, demethyleneberberine, columbamine, epiberberine, coptisine, jatrorrhizine, berberrubine, palmatine and berberine were purchased from Chroma-Biotechnology Co., Ltd. (Chengdu, China). Their purities are higher than 98%. AChE and phosphate buffered saline were purchased from Sigma-Aldrich Shanghai Trading Co. Ltd. (Shanghai, China). 5,5’-Dithiobis-(2-nitrobenzoic acid) (DTNB), S-Acetylthiocholine iodide (ATCHI) and sodium dodecyl sulfate (SDS) were purchased from Adamas Reagent Co., Ltd. (Shanghai, China). Methanol, acetonitrile and formic acid of chromatographic grade were obtained from Thermo Fisher Scientific (Shanghai, China). Deionized water was produced by an ultrapure water system (Millipore, USA). Other solvents such as n-butanol, ethyl acetate petroleum ether and dimethylsulfoxide (DMSO) were analytical grade and came from Kelong Chemical Co., Ltd. (Chengdu, Shanghai).

### Accelerated solvent extraction

Accelerated solvent extraction was achieved by a Speed Extractor E-916 instrument (BÜCHI, Switzerland) according to the developed method (Chen et al., 2021). Briefly, 1 g per sample of RC drugs was weighed and placed in a 40-mL extraction cell mixed with approximately 50 g quartz sand. A series of extracted fractions of petroleum ether, ethyl acetate and n-butanol (water-saturated) with different polarities sequentially set, with temperatures and pressures of 90°C and 100 bar, respectively. Finally, the collected extracts were evaporated by a rotary evaporator. The fractions of petroleum ether and ethyl acetate were redissolved in 10% dimethylsulfoxide at a concentration of 0.2 g/mL, while the fractions of water-saturated n-butanol were redissolved in 50% methanol at a concentration of 0.2 g/mL. Related parameters of this technique were exhibited in **Table S1**.

### Evaluation of AChE inhibitory activity

An *in vitro* AChE inhibition test was conducted based on a previous report with slight modification (Kaufmann et al., 2016). The experiment was performed in a 96-well microplate and mainly included three parts: 1) Sequentially adding AChE (0.1 U/mL) and RC extracts into phosphate buffer; 2) Combining DTNB (2.5 mmol/mL) and ATCHI (10 mmol/mL) for the reaction at 37°C for 10 min; 3) Prohibiting the reaction at 37°C for 10 min using SDS (1%) solutions. After the reaction, a SpectraMax iD3 microplate reader (Molecular Devices, USA) was applied to measure the absorbance at 405 nm. The inhibition (%) ratio was calculated by the following formula: Inhibition (%) = [1-(AB*_a_*-AB*_b_*)/(AB*_c_*-AB*_d_*)]×100% (where AB*_a_* is the absorbance of the mixture with sample and AChE; AB*_b_* is the absorbance of the mixture only with sample solution; AB*_c_* is the absorbance of the mixture only with AChE; AB*_d_* is the absorbance of the mixture without both sample solution and AChE). The same volume of phosphate buffer was used to make up the missing solutions. All samples were analysed in triplicate. The IC 20, IC 50 and IC 80 values were calculated using a logistic regression method to display the anti-AChE activity of different extracts. Prior to the sample determination, the AChE inhibition activity of the blank reconstitution solvent was determined to ensure the accuracy of the final results. Detailed operation process was exhibited in **Table S2**.

### UPLC/QqQ-MS analysis

A UPLC/QqQ-MS system with a Waters ACQUITY UPLC H-Class connected online to a Waters Xevo Triple quadrupole (TQD) mass spectrometer (Waters, Milford, MA, USA) was applied to determine the alkaloid profiles of RC extracts according to our developed method (Zhong et al., 2020). The extracts were chromatographically separated by an ACQUITY UPLC BEH C18 (100 × 2.1 mm, 5 m) with a column temperature of 25°C and a flow rate of 0.4 mL/min. The mobile phases were 0.1% formic acid water (A) and acetonitrile (B). The following gradient program was set: 0-2 min, 85%-76% A; 2-6 min, 76%-75.5% A; 6-8 min, 75.5%-75.4% A; and 8-10 min, 75.4%-75% A. The injection volume and detection wavelength were set as 1 L and 320 nm, respectively.

The Xevo TQD mass spectrometer was conducted in positive ion mode. High purity nitrogen and helium was applied as nebulizing gas and collision gas, respectively. The mass spectrometry conditions are listed below: capillary voltage: 2.5 KV; cone voltage: 25 V; source temp: 120°C; desolvation temp: 500°C; desolvation gas: 1000 L/hr; cone gas: 50 L/hr; Full scan range: 100-1200 amu; scan mode: MSe. The RC extracts were determined based on the multiple reaction monitoring (MRM) mode and the cone voltages and collision energies were optimized according to the alkaloid reference standards.

### Spectrum-effect relationship analysis

Spectrum-effect relationship analysis was applied to interpret the role of the main alkaloids, which explained the anti-AChE bioactivity of RC extracts by defining the AChE inhibition rate as the dependent variable “*Y*” and defining the alkaloid profiles as the independent variable “*X*”. Because data processing methods have an important influence on spectrum-effect relationship results, we combined the random forest (RF), Boruta and Pearson correlation based on different mathematical principles to obtain a more accurate result.

RF is an ensemble machine learning method that addresses relationships between abundant “*X*” variables and “*Y*” response in a high-dimensional space (Speiser et al., 2019). For this algorithm, a set number of trees (*n_tree_*) that are independent of one another is selected as an individual classifier. Next, a random subspace of variables (*m_try_*) regarding each individual classifier was defined to minimize the model error. An increase in mean squared error (*IncMSE*) based on the permutation importance represents the importance of each variable in the RF model. By randomly assigning a value to each variable, the error of the RF model will increase if this variable is important (Kursa and Rudnicki, 2010). Based on the RF model, Boruta (Altmann et al., 2010) introduces the shadow attribute to evaluate the importance of each “*X*” variable to explain the dependent variable “*Y*”. The shadow attributes are used as a reference to calculate the Z score to decide which variables are truly important. Pearson correlation (Yang et al., 2021) is a method to measure the linear correlation of two variables. The correlation coefficient is between 0 and 1 and represents the relevance between two variables. A numerical value of 1 means that the two variables are positively correlated, while a coefficient value of 0 means that the two variables are negatively correlated.

Finally, the *IncMSE*, Z score and Pearson coefficient were output to interpret the importance of each alkaloid responsible for the anti-AChE activity based on the developed spectrum-effect relationship model. We performed fuzzy aggregation connective operators (minimum, maximum, average and product) to make the fusion for the outputs from three different mathematical algorithms (Obisesan et al., 2017). For the three highest ranked attributes, the multiple regression technique was used to construct the linear equation to directly exhibit the relationship between alkaloids and AChE inhibition activity.

### Simulation of molecular docking

Molecular docking aims to search for active small molecules with binding potency to protein receptors based on the complementary laws of geometry, energy and chemical environment (Mtemeli et al., 2022). According to the minimum binding free energy principle, the scoring function ranks the binding ability between ligands and receptors, which represents the bioactivity of small molecules. This high-throughput technique performs at a virtual level without wasting solvent and monomer components and has been extensively applied to screen bioactive molecules from herbal medicines.

The crystal structure of AChE in complex with donepezil (PDB ID: 6O4W, resolution: 2.35 Å) (Gerlits et al., 2019) was downloaded from the RCSB PDB database. The three-dimensional structures of alkaloids were obtained from the SciFinder database. AutoDock Vina programs (Eberhardt et al., 2021) were applied to perform the molecular docking model. For the molecular docking process, we selected co-crystallized ligands of donepezil for AChE as the reference to ensure the accuracy of the active site. The molecular docking process is briefly described as follows: 1) optimizing the receptor file by deleting water molecules, adding hydrogen and charges, merging nonpolar hydrogen atoms, calculating atomic local charges, etc., and saving it in the pdbqt format; 2) optimizing the ligand files by adding hydrogen and charge and saving it as a pdbqt format file; 3) setting the number of grid points and coordinates of the centre point of the docking and saving it as a GPF file; 4) performing the molecular docking and analysing the interaction between receptor and ligands. The related tools and functions are detailed in **Table S3**.

## Results

### Anti-AChE potency of different extracts for four RC drugs

**Figure 2:**
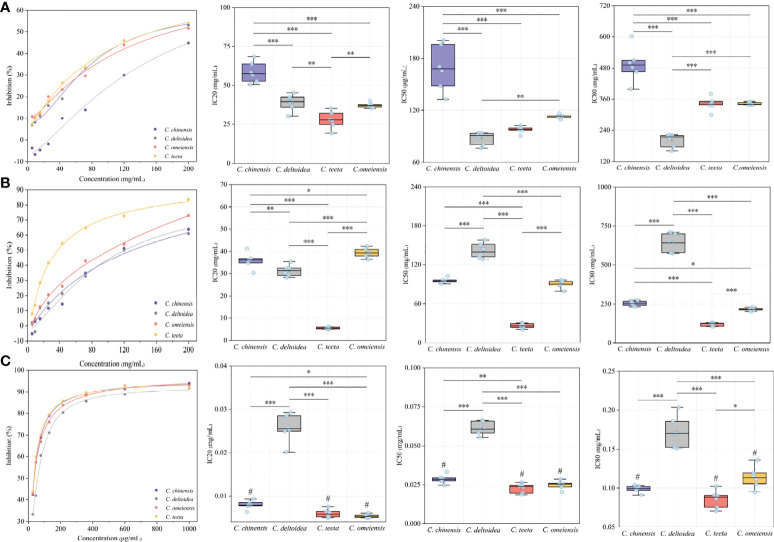
The results of *in vitro* inhibitory assays. This figure presents the AChE inhibitory curve and their IC 20, IC 50 and IC 80 of petroleum ether, ethyl acetate and n-butanol fractions of RC drugs for four *Coptis* species. The inhibitory curves were constructed by diluting the test solution in a 60% gradient. The letters of **(A–C)** represent the inhibitory of petroleum ether, ethyl acetate and n-butanol fractions of different RC drugs, respectively. The mark of “#“ means the AChE inhibition of this extract is stronger than that of positive drug. The meaning of *, **, *** symbol is P<0.05, P<0.01, P<0.001, respectively.

Natural AChE inhibitors have recently attracted more interest for the treatment of the symptoms of Alzheimer’s disease. Three petroleum ether, ethyl acetate and n-butanol fractions of RC drugs from four *Coptis* species of *C. chinensis*, *C. deltoidea*, *C. omeiensis* and *C. teeta* were initially obtained using an accelerated solvent extraction technique. Inhibitory assays were conducted *in vitro* to evaluate the anti-AChE properties of different extracts. The results are shown in **Figure 2**. The petroleum ether and ethyl acetate fractions show only weak inhibition with IC 50 values of 76.25-200.59 mg/mL and 20.36-158.26 mg/mL, respectively (**Figures 2A, B**).

The n-butanol fraction exhibits the best AChE inhibition activity, which is more than a thousand times stronger than the petroleum ether and ethyl acetate fractions (**Figure 2C**). Previous studies have suggested that this fraction mainly contains isoquinoline alkaloids that contribute to a better AChE inhibition activity (Zhao et al., 2016; Cao et al., 2018; Lin et al., 2020a). Additionally, the AChE inhibition activity of RC drugs from different *Coptis* species greatly varies. The RC drug from *C. teeta* exhibits the best inhibition activity with a significantly lower IC 50 (22.88 ± 3.05 g/mL) than *C. chinensis* (28.64 ± 2.79 g/mL, P<0.01) and a significantly lower IC 80 (85.95 ± 11.56 g/mL) than *C. omeiensis* (113.86 ± 13.69 g/mL, P <0.05). The RC drugs from *C. chinensis* and *C. omeiensis* exhibit considerable inhibitory activity, with not significantly different IC 50 (28.64 ± 2.79 g/mL vs. 24.91 ± 2.72 g/mL) and IC 80 (98.90 ± 4.40 g/mL vs. 113.86 ± 13.69 g/mL). However, IC 20 between these RC drugs presents a significant variation (7.89 ± 0.98 g/mL vs. 5.35 ± 0.42 g/mL, P<0.05). Comparatively, the RC drugs from *C. deltoidea* show the weakest inhibitory activity, with an extremely significant variation compared to the drugs from the other three *Coptis* plants (P<0.001).

We also determined the anti-AChE activity of huperzine A, which is used as a positive control drug to treat Alzheimer’s disease. Its IC 20, IC 50 and IC 80 are 22.63 ± 1.52 g/mL, 74.05 ± 15.26 g/mL and 177.25 ± 8.47 g/mL, respectively. The AChE inhibition activity of huperzine A is comparable to the N-butanol fraction of RC drugs from *C. deltoidea*. The N-butanol fraction of RC drugs from the other three *Coptis* species manifests a much stronger anti-AChE potential than huperzine A according to our *in vitro* inhibition assay.

### Quantitative analysis of the main alkaloids in n-butanol extracts

The n-butanol fraction of RC drugs exhibits the best anti-AChE property. In our previous research, we qualitatively identified 15 alkaloids, which accounted for most chemical constituents in RC drugs (Chen et al., 2021). Herein, we focused on the precise quantification of 10 common alkaloids in the n-butanol fraction based on the MRM mode of UPLC/QqQ-MS technique. For each compound, a calibration curve was constructed by plotting its peak area against the standard concentration (**Table 1**). Other methodological parameters are also optimized using reference standards and exhibited in **Tables S4, S5**. These results indicate that the developed UPLC/QqQ-MS method can be used to simultaneously and precisely determine 10 alkaloids in the n-butanol fraction of RC drugs.

**Table 1:** The methodological parameters of the UPLC−MS/MS method.

Alkaloid	Standard	Linear Interval	Correction	Limit of	Limit of
Compounds	Curves	(g/mL)	Coefficient	Detection	Quantitation	
				(g/mL)	(g/mL)
Magnoflorine	y=4772370.39x+190.05	2.28-39.48	0.9998	0.24	0.81
Groenlandicine	y=13875850.89x-5361.91	0.44-45.64	0.9996	0.36	1.21
Demethyleneberberine	y=134061x+39.415	0.068-3.19	0.9996	0.04	0.09
Columbamine	y=31008479.81x-1094.99	1.69-42.00	0.9999	0.37	1.22
Epiberberine	y=19062767.74x+2286.64	1.21-86.80	0.9999	1.00	3.32
Coptisine	y=19040,809.50x-273.95	3.99-98.82	0.9999	0.87	1.53
Jatrorrhizine	y=14793030.39x+696.38	2.07-51.2	0.9999	0.48	1.61
Berberrubine	y=31230x-38.964	1.39-40.35	0.9991	0.11	0.34
Palmatine	y=19209155.01x+6732.86	3.13-77.60	0.9998	0.97	3.22
Berberine	y=18878818.98x-8645.61	38.71-470.00	0.9998	6.93	23.9

**Figure 3:**
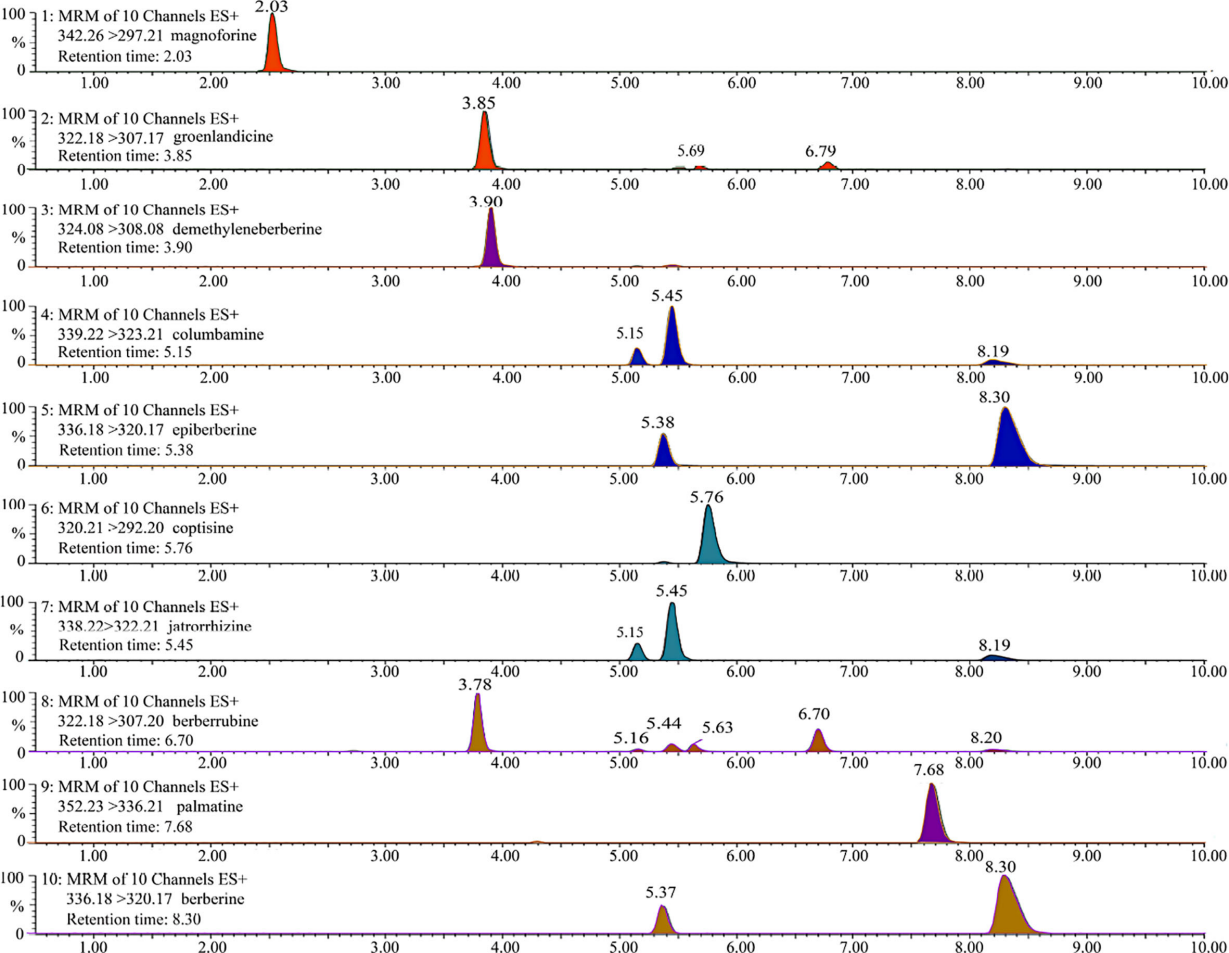
The representative MRM profiles of 10 alkaloids in RC drugs based on UPLC/QqQ-MS technique. These compounds were detected in positive ion mode, and all target alkaloids are excellently separated according to their retention time and ion fragment characteristics.

**Figure 4:**
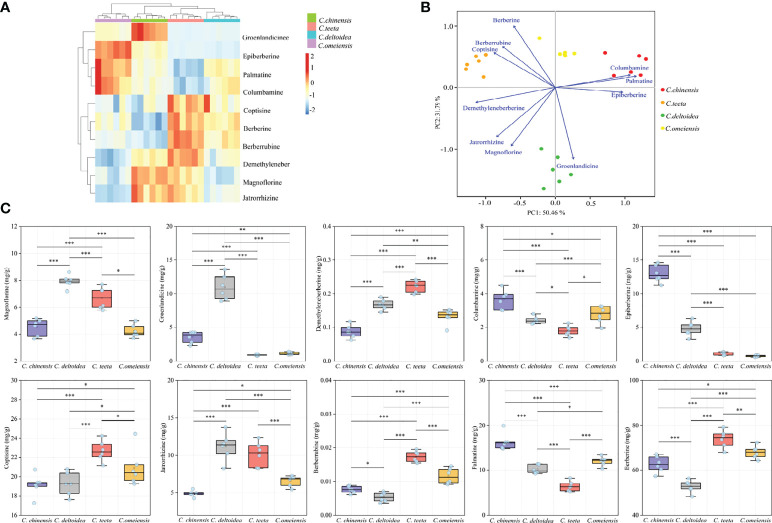
The quantitative results of 10 alkaloids in RC drugs. **(A)** hierarchical clustering presents the variation of 10 alkaloids in different RC drugs; **(B)** biplot displays the relationships among scores and loadings of classification model; **(C)** the specific content of each alkaloid. The meaning of *, **, *** symbol is P<0.05, P<0.01, P<0.001, respectively.

The UPLC chromatogram is presented in **Figure S1**. The MRM profiles of 10 alkaloids are shown in **Figure 3**. Combining their retention time and ion fragment characteristics, all target alkaloids are excellently separated and detected in the n-butanol fraction of RC drugs from different *Coptis* species. Based on the calibration curves, their concentrations were calculated. The hierarchical clustering algorithm holistically presents an excellent separation among four RC drugs from different *Coptis* species, and this result demonstrates that their concentrations of 10 alkaloids are apparently different (**Figure 4A**). Biplot simultaneously displays the relationships among scores and loadings of classification models of different RC drugs (**Figure 4B**). Coptisine and berberrubine can be used as identification markers for RC drugs from *C. teeta*, and groenlandicine can be used as an identification marker for RC drugs from *C. deltoidea*. Regarding RC drugs from *C. chinensis*, palmatine and columbamine are more valuable indicators to discriminate these drugs from others.

**Figure 4C** specifically shows the distribution of 10 alkaloids in RC drugs from different *Coptis* species. Berberine (48.27-79.26 mg/g) is identified as the most accumulated compound in RC drugs, followed by coptisine (17.26-24.47 mg/g), palmatine (5.26-19.98 mg/g), jatrorrhizine (4.25-13.76 mg/g) and epiberberine (0.61-14.61 mg/g). These conclusions are consistent with the previous papers (Zhong et al., 2018; Qi et al., 2018). In addition, RC drugs from *C. teeta* accumulate the highest berberine (73.95 ± 3.96 mg/g), followed by drugs originating from *C. omeiensis* (68.03 ± 2.82 mg/g) and *C. chinensis* (62.69 ± 3.63 mg/g). This compound in RC drugs from *C. deltoidea* (52.75 ± 2.78 mg/g) is extremely low compared with other medicinal materials (P<0.001). RC drugs from *C. teeta* also synthesized the highest *coptisine*, which was significantly higher than other RC drugs (P<0.05). This conclusion is inconsistent with previous research that RC drugs from *C. chinensis* have the highest coptisine (Chen et al., 2017). This variation may be caused by the difference in extraction techniques. The trace compounds demethyleneberberine (0.22 ± 0.02 mg/g, P<0.001) and berberrubine (0.02 ± 0.002 mg/g, P<0.001) also accumulated the most in RC drugs from *C. teeta*. RC drugs from *C. chinensis* accumulated the highest palmatine (16.36 ± 1.88 mg/g, P<0.001), columbamine (3.65 ± 0.57 mg/g, P<0.05) and epiberberine (12.98 ± 1.28 mg/g, P<0.001) levels. In addition, magnoflorine (7.94 ± 0.46 mg/g, P<0.001), groenlandicine (10.95 ± 1.87 mg/g, P<0.001) and jatrorrhizine (11.17 ± 1.83 mg/g) were the most abundant in RC drugs from *C. deltoidea*.

Differences in chemical compound concentrations of herbal medicines cause their different therapeutic efficacies. The alkaloid variation in the four RC extracts is the main reason that explains their different AChE inhibitory activities. Furthermore, the relationship between alkaloid profiles and anti-AChE activity was investigated to reveal the detailed mechanism underlying the efficacy difference.

### Spectrum-effect analysis between anti-AChE properties and alkaloid profiles

**Figure 5:**
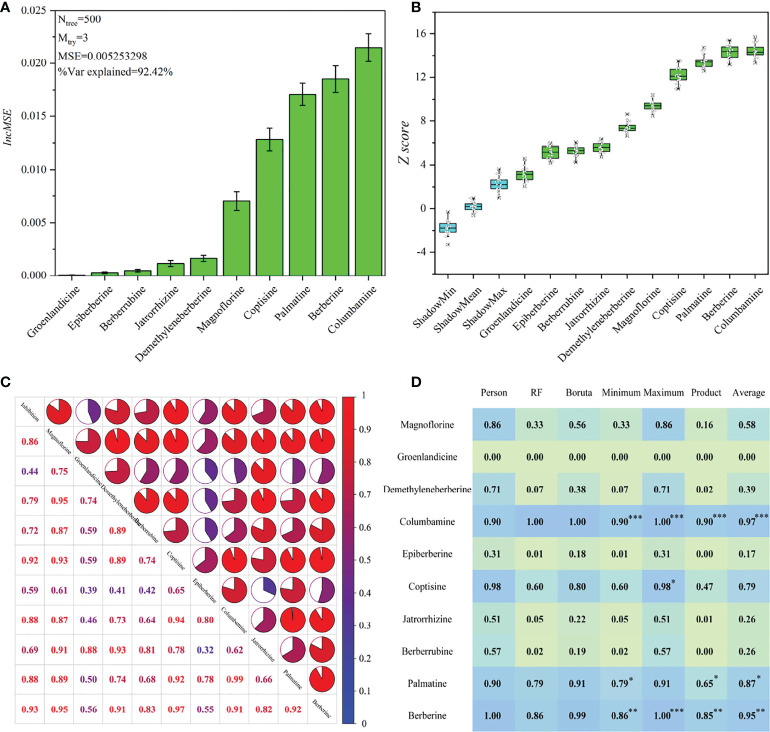
The results of spectrum-effect relationship based on different data processing methods. **(A)** the ranking result based on RF model; **(B)** the ranking result based on Boruta model; **(C)** the result of Pearson correlation analysis; **(D)** data fusion results, and ***, ** and * mean the first, second and third ranking of these components, respectively.

To obtain a convincing result, we used three data processing methods with different mathematical principles to exhibit the spectrum-effect relationship. The corresponding results are exhibited in **Figure 5**. A reliable RF model (*MSE*=0.00525) that explains 92.42% of the variables for the alkaloid matrix was first established with *n_tree_* and *m_ttry_* values of 500 and 3, respectively. The *IncMSE* score indicates that columbamine was most highly correlated with the AChE inhibitory activity, followed by berberine and palmatine (**Figure 5A**). Boruta was applied by setting shadow attributes, and the Z scores was used to represent the importance of these alkaloids. The *Z* scores of all alkaloids are higher than the shadow attributes, which indicates that they are highly correlated with AChE inhibitory activity. Comparatively, columbamine and berberine are the most important variables responsible for the anti-AChE activity (**Figure 5B**). The result from this algorithm is close to that from RF.

Furthermore, we performed a Pearson correlation analysis to exhibit the relational degree between AChE inhibition and 10 alkaloids and their own associations (**Figure 5C**). The results show that berberine and coptisine are the first two key compounds responsible for the anti-AChE activity, followed by palatine and columbamine. The results from this algorithm were obviously different from those of RF and Boruta because their internal mathematical operation methods were different. In addition, columbamine and palatine have the highest correlation (relational degree=0.99) because the former is the precursor material for the biosynthesis of the latter (Liu et al., 2022). Modern research has proved that some key enzymes required for the berberineand coptisine biosynthesis are identical, which can explain their high correlation with accumulations (relational degree=0.97) (Liu et al., 2021).

To take these correlation scores together, we made a data fusion for the minimum, maximum, average and product fuzzy aggregation connective operators based on different outputs from these data processing methods (Obisesan et al., 2017). The voting results indicate that the top three high-contributing alkaloids for AChE inhibitory activity are columbamine, berberine and palmatine (**Figure 5D**).Lin et al., (2020a) performed monomer experiments and indicated the relatively higher anti-AChE potential of berberine and columbamine. We also determined the AChE inhibition of eight alkaloid monomers. The results indicate that the inhibitory activities of these compounds were as follows: palmatine (1.49 ± 0.4 g/mL)> columbamine (2.29 ± 0.7 g/mL)> berberine (3.22 ± 0.9 g/mL)> coptisine (4.34 ± 1.5 g/mL)> groenlandicine (5.84 ± 2.0 g/mL)> jatrorrhizine (6.36 ± 2.2 g/mL)> epiberberine (10.14 ± 3.4 g/mL)> magnoflorine (21.26 ± 4.5 g/mL) (**Figure S2**). The previous published papers also showed that the AChE inhibition of demethyleneberberine and berberrubine are much weaker than berberine (Huang et al., 2010; Roselli et al., 2016). These results proved that the spectrum-effect analysis was effective in interpreting the relationship between AChE inhibition activity and alkaloids. All tested alkaloids have much lower IC 50 than huperzine A, which indicates the huge anti-AChE potential of these alkaloids. Finally, the multiple regression technique presented the linear equation of AChE inhibition (YAChE) with berberine (X*_b_*), columbamine (X*_c_*) and palmatine (X*_p_*). After removing insignificant coefficients, the formula is exported as YAChE =0.60521X*_b_* +3.3591X*_c_*-1.9141X*_p_* +36.878 $X_{c}^{2}$ -72.817X*_c_* X*_p_* +34.125 ${\text{X}}_{p}^{2}$ +0.1297, R^2^=0.94. All model parameters are significant, revealing that the fitting effect of this equation is excellent (**Tables S6, S7**).

### Molecular docking

To obtain better insight into the properties of columbamine, berberine and palmatine against AChE, molecular docking was performed to analyse their ligand-active site interactions. Searching the effective active site in the receptor for drug candidates with a low binding energy is a critical step for molecular docking (Feinstein and Brylinski, 2015). The centre coordinates (*X*: 94.58; *Y*: 94.72; *Z*: 17.01) of the docking box for AChE protein were optimized according to the location of the co-crystallized ligand of donepezil (Brewster et al., 2018). The three-dimensional structures of these alkaloids after energy minimization are exhibited in Fig S3. AutoDock Vina software was used to perform molecular docking, and the first docking poses were output according to the rank of scoring function.

**Figure 6:**
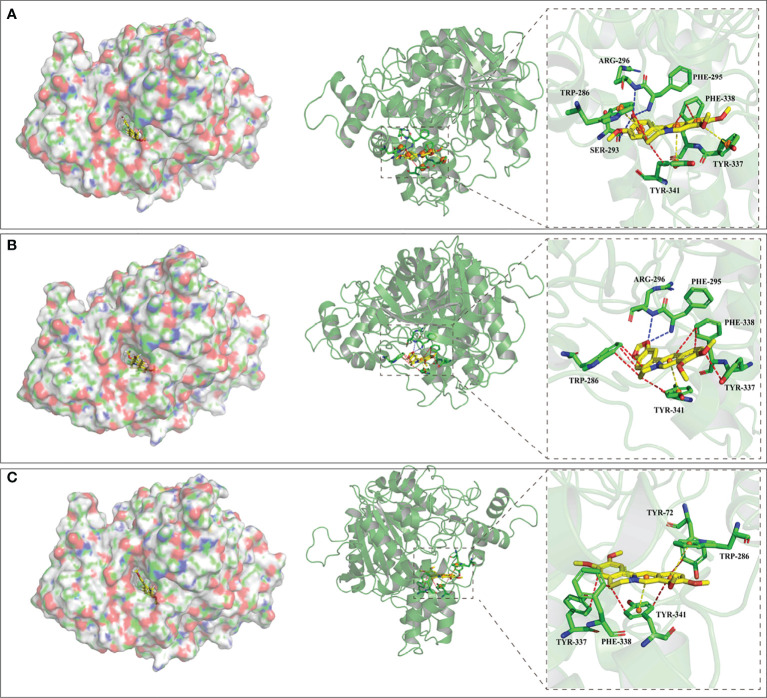
The visualization of molecular docking results. The letters of **(A–C)** represent the visualization result columbamine, berberine and palmatine, respectively. The left parts exhibit these compounds interact with AChE at a long andnarrow hydrophobic pockets, and the rights parts exhibit the detail of these interactions. Red, blue and yellow lines represent hydrophobic interaction, hydrogen bond and - stacking interaction, respectively.

**Table 2** exhibits binding affinities, including hydrogen bonds, hydrophobic interactions and pi-pi interactions, which ensure stable interactions between alkaloids and AChE. The *in vitro* results show that the binding energies of columbamine, berberine and palmatine are -9.9, -10.6 and -9.8 Kcal/mol, respectively, which indicates that these small molecules can dock with AChE in a natural state. The visualized images are presented in **Figure 6**. All of these bioactive components interact with enzyme crystals at long and narrow hydrophobic pockets, which is similar to donepezil. Tyr-337 and Trp-286, which can maintain the geometry of the binding gorge and provide electrostatic balance, are important residues of the AChE protein (Kua et al., 2003). Columbamine, berberine and palmatine can interact with these residues *via* hydrophobic and - stacking interactions. Additionally, columbamine is stabilized by hydrophobic interactions with Tyr-341, Trp-286 and Phe-338, hydrogen bonds withPhe-295, Ser-293 and Arg-296, and - stacking interactions with Tyr-341 (**Figure 6A**). Berberine is stabilized by hydrophobic interactions with Tyr-341 and Phe-338, hydrogen bonds with Phe-295 and Arg-296, and - stacking interactions with Tyr-341 (**Figure 6B**). Palmatine presents three strong hydrophobic interactions with Tyr-341, Tyr-72 and Phe-338 and a - stacking interaction with Tyr-341 (**Figure 6C**).

**Table 2:** Binding sites and action forces between components and AchE protein.

Ligand Molecules	Binding Energy (Kcal/mol)	Hydrophobic Interactions	Hydrogen Bonds	π-π stacking Interaction
Columbamine	-9.9	TRP-286, PHE-338, TYR-341	SER-293, PHE-295, ARG-296	TRP-286, TYR-337, TYR-341
Berberine	-10.6	TRP-286, TYR-337, PHE-338, TYR-341	PHE-295, ARG-296	TYR-341
Palmatine	-9.8	TYR-72, TYR-337, PHE-338, TYR-341	–	TRP-286, TYR-341
Donepezil	-6.61	TRP-86, TYR-337, PHE-338, PHE-341	PHE-295, TYR-337	TRP-86, TRP-286, TYR-341

Based on X-ray crystallography in previous study (Gerlits et al., 2019), the specific reversible central AChE inhibitor of donepezil can interact with this receptor by forming stacking interactions, hydrophobic interactions and hydrogen bonds with amino acid residuesTrp-86, Trp-286, Tyr-337, Phe-338, Tyr-341, Ser-293, Phe-295, and Tyr-72. Our docking results show that columbamine, berberine and palmatine mainly interact with these residues to dock towards the AChE protein. These computer simulation results explain the molecular mechanism of columbamine, berberine and palmatine against AChE, which is also consistent with the *in vitro* assay. In summary, these components are potential AChE inhibitors and can be considered quality markers to evaluate the anti-AChE activity of RC drugs from different *Coptis* species.

## Discussion

Acetylcholine deficiency is an important factor that promotes the occurrence and progression of Alzheimer’s disease. AChE is a serine hydrolase that can terminate nerve impulse transmission by transforming acetylcholine into acetate and choline in the central and peripheral nervous systems (Stanciu et al., 2019). Considering the side effects of current drugs for AChE inhibition, it is necessary to screen natural AChE inhibitors with fewer toxic side effects to treat neurological diseases. Natural products have a long application for the treatment of many ailments, and the ingredients originally isolated from them are relatively safe (Guan et al., 2017; Abate et al., 2021; Wang et al., 2022).

**Figure 7:**
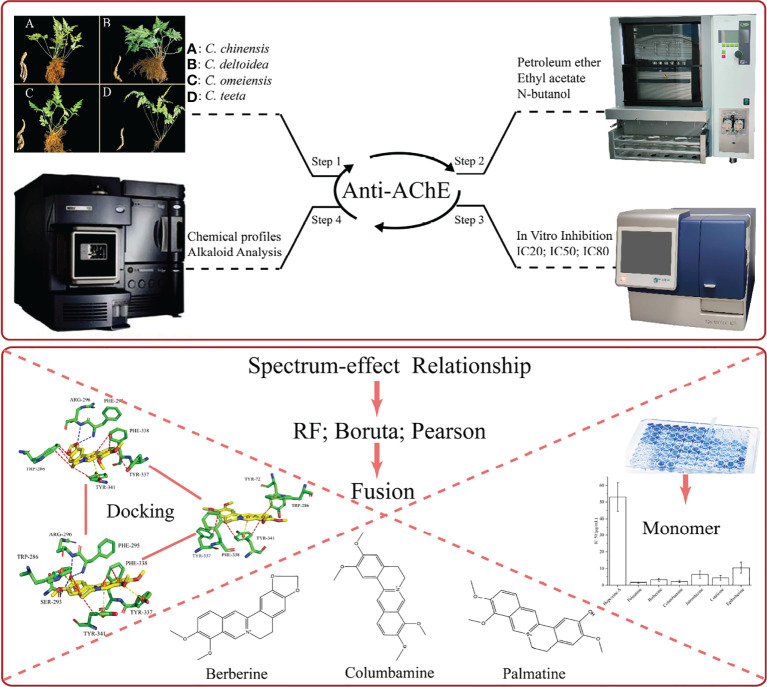
The overall analysis strategy of this article. The above section mainly describes the experimental process, and the following section mainly describes the analysis process.

The roots and rhizomes of *Coptis* species have been used as RC drugs for thousands of years in traditional oriental medicine. Several ancient pharmacological writings in China record that these drugs can effectively treat brain diseases. Currently, RC drugs have been clinically applied to prevent and treat Alzheimer’s disease. The investigation regarding the AChE inhibitory activity of *Coptis* species is insufficient. Thus, we designed an analysis strategy to investigate for the first time the anti-AChE potential of different extracts for RC drugs from four Coptis species and to analyse the role of the main alkaloids on this potency (**Figure 7**).

*In vitro* inhibitory assays proved that n-butanol was the optimized fraction for AChE inhibition, which is consistent with the previous studies (Zhao et al., 2016; Cao et al., 2018; Lin et al., 2020a). *C. teeta* can be used as a better source of RC drugs than the commonly used *C. chinensis* due to its relatively high AChE inhibition. Meanwhile, we focused on the rapid absolute quantitation of 10 common alkaloids in RC drugs based on our previous qualitative research. Compared with conventional liquid chromatography, the MRM mode of UPLC/QqQ-MS has excellent selectivity and sensitivity for these target compounds (Li et al., 2021). The result showed that these alkaloids in different drugs greatly varied. We also identified several trace compounds as markers to discriminate RC drugs from different sources, such as groenlandicine and magnoflorine for RC drugs from *C. deltoidea*, and berberrubine and demethyleneberberine for RC drugs from *C. teeta*. Overall, these findings can provide scientific evidence for the application of RC drugs to treat brain diseases.

Many studies have demonstrated that spectrum-effect relationship and molecular docking are powerful tools to interpret the mechanism of multi-component systems in herbal medicines that exert their therapeutic effect. We performed these techniques to analyse the relationship between alkaloids and AChE inhibition of RC drugs. Three data processing methods were applied to develop the spectrum-effect model, and a data fusion strategy was used to calculate a comprehensive result. Boruta algorithm indicates the importance of 10 alkaloids for AChE inhibition activity due to their higher *Z*-score than the shadow attributes. Comparatively, columbamine, berberine and palmatine were identified as the best AChE inhibitors. Considering the concentrations of these compounds in RC drugs, berberine is the most critical factor that determines the anti-AChE potential of RC drugs from different sources. Furthermore, we constructed a linear relationship between AChE inhibition activity and the first three alkaloids. This equation may be used to guide the RC drug selection to treat Alzheimer’s disease. Compared with the co-crystallized ligands of donepezil, molecular docking identified the key binding forces between these alkaloids and the AChE protein at the mechanistic level. All of these results demonstrated that alkaloids are the substance foundation for the anti-AChE potential, and columbamine, berberine and palmatine are quality markers to evaluate this activity in clinical application.

## Conclusion

The present study developed *in vitro* inhibitory assays to investigate the anti-AChE potential of different extracts of RC drugs from *Coptis* species and the UPLC/QqQ-MS method to quantify the main alkaloids in the n-butanol fraction. Spectrum-effect relationship and molecular docking were applied to interpret the associations between these alkaloids and AChE inhibitory properties. Columbamine, berberine and palmatine were screened and identified as the quality markers responsible for the AChE inhibition activity. We hope that these results can contribute to the effective development and utilization of RC drugs from different sources, especially to provide certain evidence for their application in the treatment of Alzheimer’s disease. Of course, the clinical application of these medicinal materials warrants further validation.

## Data availability statement

The original contributions presented in the study are included in the article/**Supplementary Material**. Further inquiries can be directed to the corresponding authors.

## Author contributions

LQ conceived and designed the research and wrote the manuscript. FZ and NL collected the experimental materials. JW, KN and YT performed the experiments. LX and YM revised the manuscript. All authors contributed to this article and approved the submitted version.

## Funding

This work were financially supported by the National Natural Science Foundation of China (U19A201).

## Conflict of interest

The authors declare that the research was conducted in the absence of any commercial or financial relationships that could be construed as a potential conflict of interest.

## Publisher’s note

All claims expressed in this article are solely those of the authors and do not necessarily represent those of their affiliated organizations, or those of the publisher, the editors and the reviewers. Any product that may be evaluated in this article, or claim that may be made by its manufacturer, is not guaranteed or endorsed by the publisher.
