# Supplementary material for: Characterization of the anti-AChE potential and alkaloids in Rhizoma Coptidis from different Coptis species combined with spectrum-effect relationship and molecular docking
Source: Front Plant Sci. 2022 Oct 31;13:1020309. doi: 10.3389/fpls.2022.1020309 (PMC9659949; doi:10.3389/fpls.2022.1020309)
Supplement: Supplementary file 1 [file DataSheet_1.docx]

Table S1 Parameter settings of E-916 accelerated solvent extraction system

| **Solvent** | **Temperature** | **Pressure** | **Cycle** | | | **Solvent flushing** | **Gas flushing** |
| --- | --- | --- | --- | --- | --- | --- | --- |
|  |  |  | **Heating** | **Keep** | **Release** |  |  |
| Petroleum  ether | 90°C | 100 bar | 1 min | 3 min | 5 min | 2 min | 3 min |
|  |  |  | 1 min | 3 min | 5 min |  |  |
|  |  |  | 1 min | 3 min | 5 min |  |  |
| Ethyl  acetate | 90°C | 100 bar | 1 min | 3 min | 5 min | 2 min | 3 min |
|  |  |  | 1 min | 3 min | 5 min |  |  |
|  |  |  | 1 min | 3 min | 5 min |  |  |
| N-butanol  (Water saturated) | 90°C | 100 bar | 1 min | 0 min | 5 min | 2 min | 3 min |
|  |  |  | 1 min | 3 min | 5 min |  |  |
|  |  |  | 1 min | 3 min | 5 min |  |  |
|  |  |  | 1 min | 3 min | 5 min |  |  |

(Note: This table presents the main parameter settings of E-916 accelerated solvent extraction system. These parameters were selected according to our pervious which has cited in the manuscript.)

Table S2 Detailed operation process of AChE inhibition array

| **Setting** | **Buffer (μL)** | **Extraction (μL)** | **AChE (μL)** | **DNTB (μL)** | **ATCHI (μL)** | **SDS (μL)** |
| --- | --- | --- | --- | --- | --- | --- |
| AB_a_ | 100 | 0 | 20 | 40 | 40 | 60 |
| AB_b_ | 120 | 0 | 0 | 40 | 40 | 80 |
| AB_c_ | 80 | 20 | 20 | 40 | 40 | 60 |
| AB_d_ | 100 | 20 | 0 | 40 | 40 | 60 |

(Note: This table presents the detailed operation process of AChE inhibition array. This experiment was conducted in a 96-well microplate and 4 micropores named AB_a_, AB_b_, AB_c_ and AB_d_ were set for calculating the inhibition rate of each sample. For each micropore, the solutions to be added and their volumes are shown in this table.)

Table S3 Information of main tools for molecular docking

| **Tools** | **Website** | **Functions** |
| --- | --- | --- |
| Protein Data Bank | http://www.rcsb.org | Downloading AchE protein |
| Scifinder | https://scifinder.cas.org | Querying alkaloid structures |
| AutoDock Vina | http://vina.scripps.edu | Performing molecular docking |
| Protein-ligand of interaction profiler | https://plip-tool.biotec.tu-dresden.de/plip-web/plip/index | Analyzing protein-ligand of interaction |

(Note: This table presents the main tools used in molecular docking programs. This process mainly contains four steps: preparing AchE protein; preparing alkaloid ligands; performing molecular docking; Analyzing protein-ligand interaction.)

Table S4 Information of main tools for molecular docking

| **Compounds** | **Parent ion** | **Daughter ion** | **Cone voltage (V)** | **Collision energy (V)** | **Polarity** |
| --- | --- | --- | --- | --- | --- |
| Magnoflorine | 342.26 | 297.21 | 58 | 21 | Positive |
| Groenlandicine | 322.18 | 307.17 | 72 | 30 | Positive |
| Demethyleneberberine | 324.08 | 308.08 | 60 | 30 | Positive |
| Columbamine | 339.22 | 323.21 | 60 | 25 | Positive |
| Epiberberine | 336.18 | 320.17 | 60 | 34 | Positive |
| Coptisine | 320.21 | 292.20 | 80 | 33 | Positive |
| Jatrorrhizine | 338.2 | 322.21 | 60 | 32 | Positive |
| Berberrubine | 322.18 | 307.20 | 75 | 30 | Positive |
| Palmatine | 352.23 | 336.21 | 60 | 32 | Positive |
| Berberine | 336.18 | 320.17 | 64 | 34 | Positive |

(Note: This table presents the mass spectrometry parameters of UPLC/QqQ-MS method. The parent ion, daughter ion, cone voltage and collision energy were optimized using reference standards.)

Table S5: The precision, repeatability and stability of UPLC/QqQ-MS method

| **Alkaloids** | **Precision** | **Repeatability** | **Stability** |
| --- | --- | --- | --- |
|  | **RSD (%) (n=6)** | | |
| Magnoflorine | 0.35 | 0.79 | 0.45 |
| Groenlandicine | 0.96 | 1.93 | 1.02 |
| Demethyleneberberine | 1.23 | 1.37 | 0.94 |
| Columbamine | 0.77 | 0.96 | 0.38 |
| Epiberberine | 0.53 | 0.78 | 0.86 |
| Coptisine | 0.76 | 0.79 | 0.26 |
| Jatrorrhizine | 0.61 | 0.69 | 0.28 |
| Berberrubine | 1.44 | 1.56 | 1.04 |
| Palmatine | 0.6 | 0.67 | 0.51 |
| Berberine | 0.65 | 0.53 | 0.95 |

(Note: This table presents the methodological parameters of UPLC/QqQ-MS method. These methodological results are optimized using reference standards.)


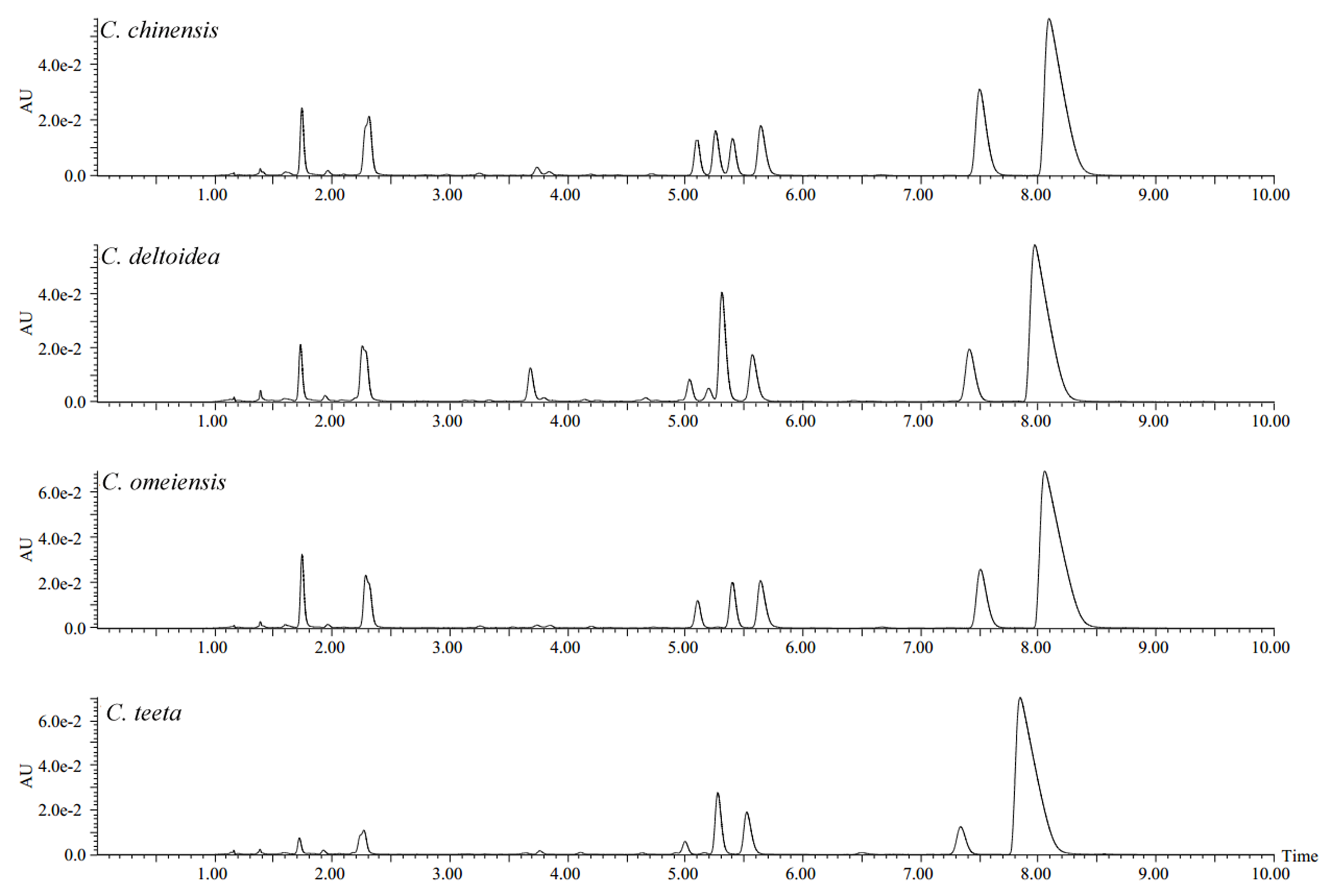


Fig S1: The UPLC chromatograms of n-butanol fraction of RC drugs from different *Coptis* species.





Fig S2: The AChE inhibition of eight alkaloid monomers and huperzine A.

Table S6: Result of multiple regression technique

| **Regression Model** | **Measurement Coefficient** | **Correction Coefficient** | **RMSE** | ***F*** | ***P*** |
| --- | --- | --- | --- | --- | --- |
| Linear regression | 0.875 | 0.868 | 0.0965 | 126 | 2.30E-24 |
| Quadratic regression | 0.954 | 0.945 | 0.0622 | 110 | 5.93E-29 |
| Stepwise regression | 0.949 | 0.943 | 0.0634 | 158 | 3.53E-31 |

(Note: This table presents the parameters of multiple regression technique used for presenting the linear equation of AChE inhibition with berberine, columbamine and palmatine. According to the results, stepwise regression was finally selected because of its high significance.)

Table S7: Parameters of stepwise regression model

|  | **Slope** | **Standard Error** | ***T*** | ***P*** |
| --- | --- | --- | --- | --- |
| Intercept | 0.1297 | 0.015506 | 8.3645 | 3.93E-11 |
| X_b_ | 0.60521 | 0.10835 | 5.5856 | 9.08E-07 |
| X_c_ | 3.3591 | 0.74643 | 4.5002 | 3.96E-05 |
| X_p_ | -1.9141 | 0.72588 | -2.6369 | 0.011061 |
| X_c_^2^ | 36.878 | 8.8849 | 4.1506 | 0.000126 |
| X_p_X_b_ | -72.817 | 17.111 | -4.2555 | 8.93E-05 |
| X_p_^2^ | 34.125 | 8.0945 | 4.2158 | 0.000102 |

(Note: This table presents the parameters of final stepwise regression model after removing insignificant coefficients. All these coefficients are highly significant, indicating the fitting effect of this equation is excellent.)


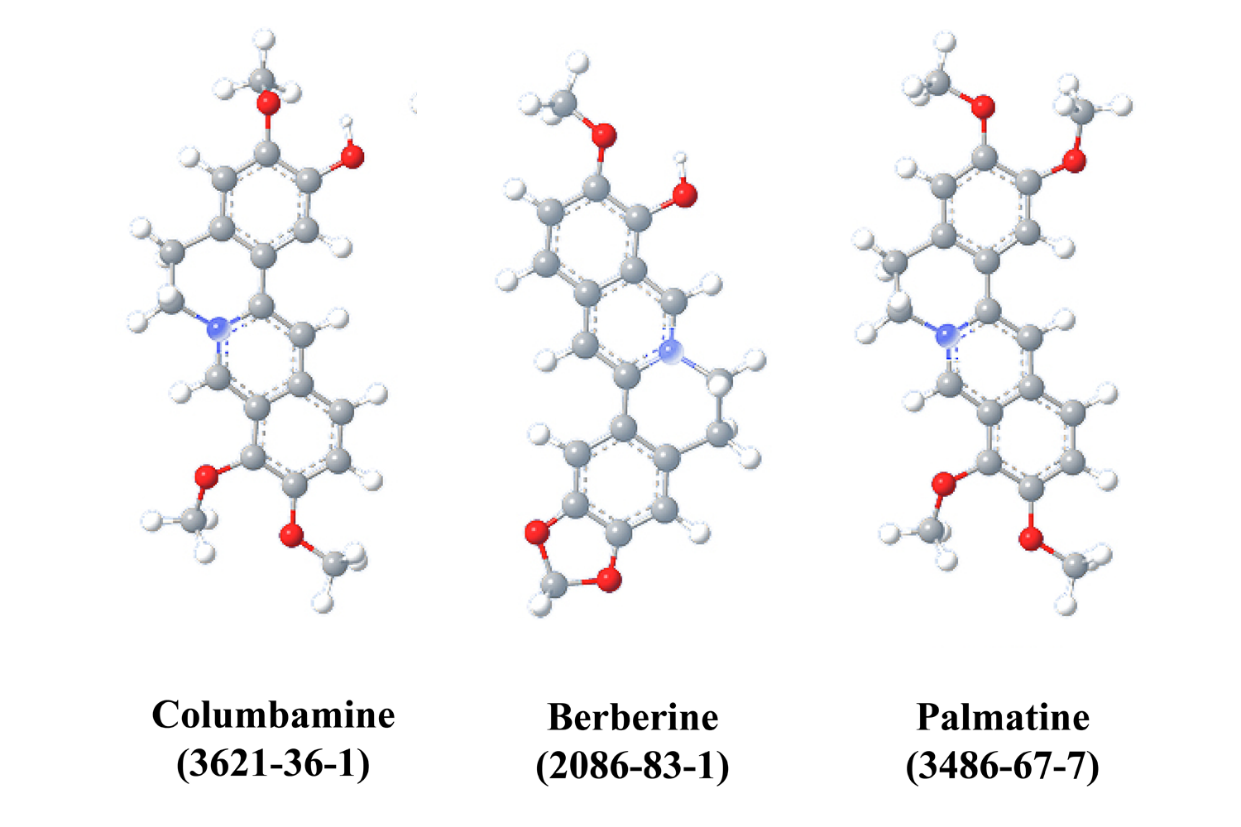


Fig S3: The three-dimensional structures of 3 alkaloid
